# The primary total knee arthroplasty: a global analysis

**DOI:** 10.1186/s13018-020-01707-5

**Published:** 2020-05-26

**Authors:** Jiaxiang Gao, Dan Xing, Shengjie Dong, Jianhao Lin

**Affiliations:** 1 Arthritis Clinic & Research Center, Peking University People’s Hospital, Peking University, Beijing, 100044 China; 2 Arthritis Institute, Peking University, Beijing, China; 3 Orthopedic department, Yantaishan Hospital, Yantai, Shandong China

**Keywords:** Total knee arthroplasty, Bibliometrics, Visualized study

## Abstract

**Background:**

The use of total knee arthroplasty (TKA) in treatment of chronic degenerative pathologies of the knee has boasted of an experience of 50 years. The aim of this bibliometric and visualized study is to comprehensively examine the current status and global trends of TKA research.

**Methods:**

Publications related to TKA research from 2010 to 2019 were retrieved from Web of Science (WoS) and Science Citation Index-Expanded (SCIE) database and then analyzed through bibliometric methodology. As for the visualized study, the software VOS viewer was utilized for bibliographic coupling, coauthorship, cocitation, and co-occurrence analysis, along with further simulation of publication trends in this field.

**Results:**

A total of 8631 publications were eventually included. The number of publications tends to increase annually worldwide. The USA was the pioneer which has made tremendous contributions, with the most publications and citations, as well as the highest H-index. *The Journal of Arthroplasty* has published the most papers, while *Clinical Orthopaedics and Related Research* has the highest citation frequency. The Hospital for Special Surgery has made the greatest contribution when total publication number and coauthorship were taken together. Studies could be divided into five clusters: “alignment study”, “revision TKA study”, “complication study”, “rehabilitation study”, and “perioperative management study”, which have a trend of balanced development in this field.

**Conclusions:**

There will be an increasing number of publications on TKA research according to the current global trends, and the USA maintained the leadership in this area. Additionally, a trend of balanced development may exist in the field of TKA research, accompanied with inherent changes of hotspots in each cluster.

## Background

Total knee arthroplasty (TKA) is considered as the most common treatment for end-stage knee osteoarthritis (OA), which also makes great sense for some other underlying indications, including inflammatory arthritis, fracture (post-traumatic OA and/or deformity), dysplasia, and malignancy [1, 2]. Contemporary TKA has evolved over the past 50 years since it was first designed at the Hospital for Special Surgery in the early 1970s [3]. Nowadays, the global demand for TKA is increasing at a dramatic rate due to the growing prevalence of knee arthritis. The number of primary TKA procedures is projected to grow by 85% (1.26 million procedures) by 2030 [4].

In recent years, TKA is experiencing unprecedented development. The collaboration between surgeons and engineers produced many developments in the design of the prosthesis, from the traditional resurfacing prostheses to the constrained prostheses and meniscal bearing prostheses [5]. Furthermore, various new technology-based assistive techniques (such as the robots, patient-specific cutting guides, preoperative software, and computer-assisted navigation system) were introduced several years ago in orthopedic surgery, which have significantly improved the accuracy and reproducibility in maintaining bone resection and ligament balance [6]. In addition, other problems derived from TKA, such as prosthesis survivorship and revision surgery, as well as the management of post-operative complications and rehabilitation, are also significant topics worthy of further attention. To our best knowledge, the global research trend in TKA has not been well studied yet. Therefore, it is necessary to investigate the global status of TKA.

Publications are critical indicators of research trends which can represent the importance of a certain field. Bibliometric analysis is a feasible method to predict the developing trends of the research qualitatively and quantitatively, by comparing the studies of main authors, journals, institutes, and nations [7]. Besides, it also makes great contributions to clinical policy-making and guidelines regulation [8]. In addition, bibliometric analysis makes it more transparent for scientific researching in different areas [9]. The aim of this study was to comprehensively examine the current status and global trends of TKA studies in different clinical areas.

## Methods

### Data source

Although a large number of databases could meet the requirement for evaluation research at a global level [10–12], we selected the Web of Science (WoS) and Science Citation Index-Expanded (SCIE) for this analysis, which cover more than 12,000 scientific international journals of the highest impact and quality, providing comprehensive data of publications [13].

### Search strategy

In order to search for the studies on TKA, the exclusion criteria should cover the most common terms related to the arthroplasty on other joints (such as hip, ankle, and shoulder joints), and other types of knee surgeries (such as osteotomy and arthroscopic surgery). The search strategy was as follows: (theme=total knee arthroplasty Not theme=hip Not theme=ankle Not theme=shoulder Not theme=lumbar Not theme== Osteotomy Not theme= arthroscop* Not theme==unicompartment*). The time period of publications was focused on the latest 10 years from 2010 to 2019, and the document types only contained reviews and articles. Retrieval work was performed in the same day (on February 5, 2020) to avoid the variations due to daily updates. Informed consent was not required because the data is all secondary data without any personal information.

### Data collection

The information of all identified publications, including title, author, year of publication, nationalities, affiliations, journal, keywords and abstract, was downloaded from the WoS database and then opened by Excel 2016 (Redmond, WA). Two authors independently browsed and extracted data from the eligible publications and further analyzed the data with GraphPad Prism5 separately.

### Bibliometric analysis

The basic characteristics of publications were retrieved through the intrinsic function of WoS. The H-index is defined as the value based on a scholar or scientist who has published *H* papers, each of which has been cited by other papers for at least *H* times [14]. Therefore, the H-index can reflect the number of both publications themselves and their relevant citations and evaluate the productivity and impact of the published scientific research [15]. The relative research interest (RRI) is the number of publications in a certain field divided by all-field publications per year [16]. R software (version 3.1.3) was used to analyze the time trend of the publications. Moreover, the cumulative volume of documentation was simulated by using the logistic regression model: *f*(*x*) = *c*/[1 + *a* × exp(− *b* × (*x* − 1994))]. In this formula, the symbol *x* refers to the year, and *f*(*x*) represents the cumulative amount of papers. The time when the growth rate of publications changed from positive to negative was defined as the inflection point, which was generated using the formula *T* = ln*a*/*b* + 1994.

### Visualized analysis

VOS viewer (Leiden University, Leiden, The Netherlands) is a software tool for constructing and visualizing bibliometric networks [17]. In this research, VOS viewer was used for bibliographic coupling, coauthorship, cocitation, and co-occurrence analysis. In the network map created by VOS viewer, different nodes represented various elements such as author, country, institution, and keyword, while the size of the nodes reflected the number of the publications or frequency [18]. The links between nodes represented the relationships such as cocitation, coauthorship, or co-occurrence [19, 20]. Furthermore, the color of the node/lines reflected different clusters or years [21].

## Results

### Global publication trends

#### Number of global publications

**Fig. 1:**
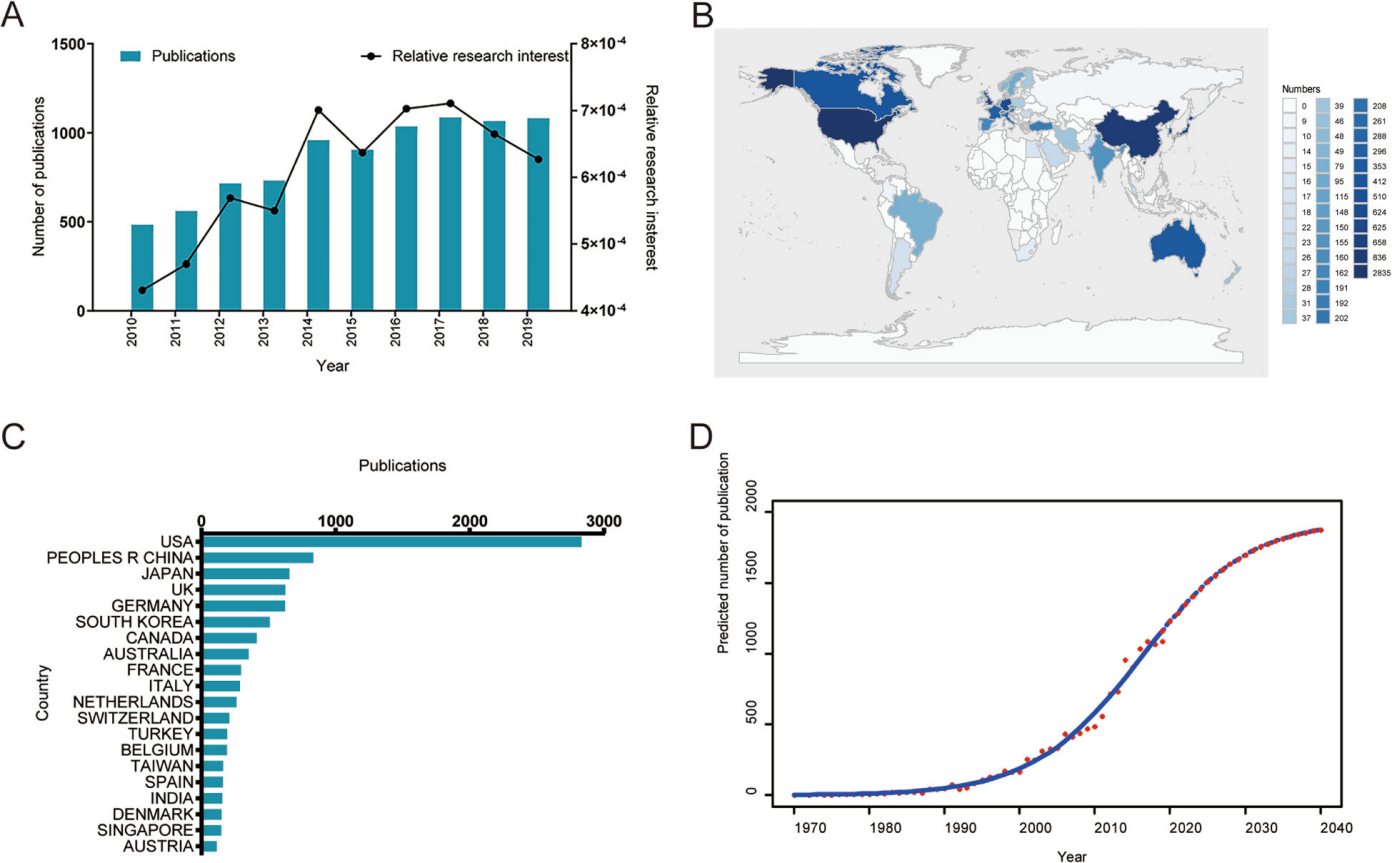
**a** The total number and RRIs of publications related to TKA research. The blue bars indicate the number of publications per year, and the black curve means the RRIs. **b** The distribution world map of TKA research. **c** The top 20 countries of total publications. **d** Model fitting curves of global publication trends

A total of 8631 publications eventually were collected after applying the search criteria. Over the past decade, the number of publications has steadily increased year by year, from 483 (2010) to 1083 (2019). In addition, most of the researches were published in 2019 (1083, 12.5%) (Fig. 1a).

#### Contributions of countries and regions

Moreover, a total of 86 countries and regions have published related articles/review, and the countries which had made the greatest contributions in TKA research were presented in Fig. 1b and c. Among them, the USA contributed to the most publications (2835, 32.8%), followed by China (835, 9.7%), Japan (657, 7.6%), and the UK (652, 7.6%).

#### Global trends of publications

In order to predict the future, the time curve of the all-year publication numbers was created by using the logistic regression model, and the model fitting curves of the growth trend was shown in Fig. 1d. Furthermore, with the help of this time curve, it is estimated that the number of the publications in this domain would grow by nearly 3.5 times from 483 in 2010 to approximately 1700 by 2030.

### Quality of the publications of each country/region

#### Total citation frequency

**Fig. 2:**
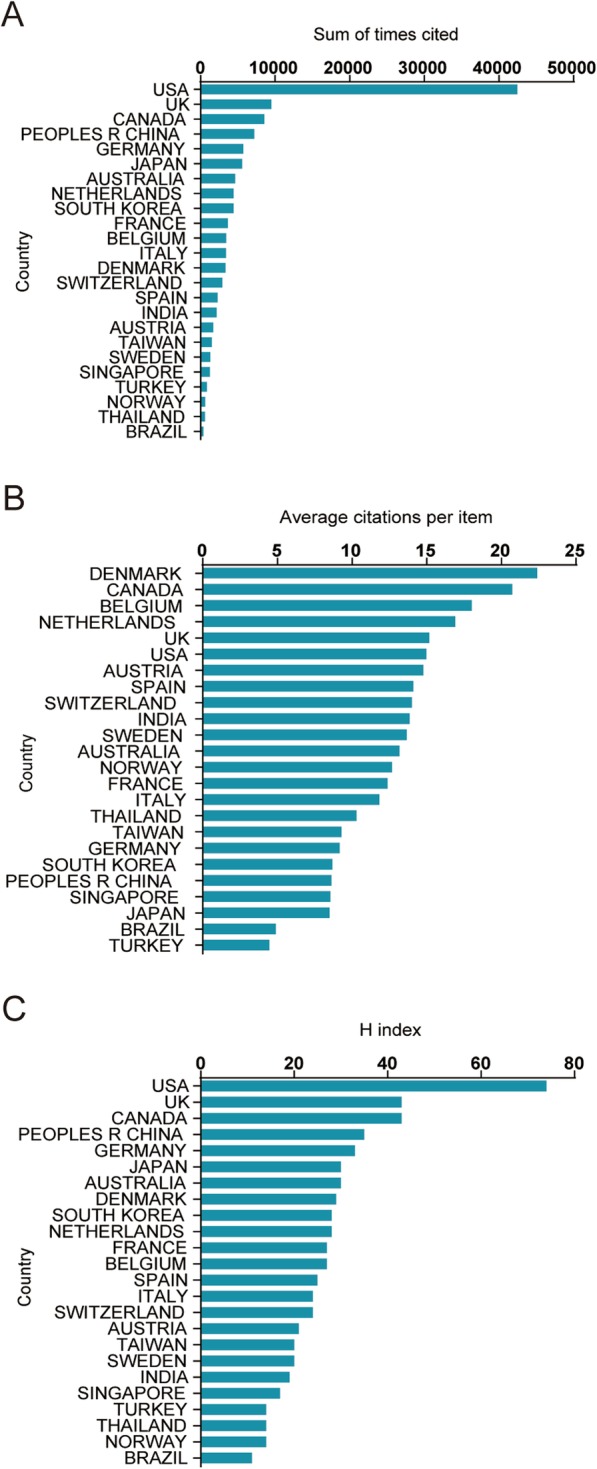
**a** The top 20 countries of total citations. **b** The top 20 countries of average citations for each article. **c** The top 20 countries of the H-index

The USA had the highest number of total citations (42,461) among all the included publications, while the UK ranked the second (9487), then followed by Canada (8541), China (7199), and Germany (5715) (Fig. 2a).

#### Average citation frequency

Publications from Denmark had the most average number of citations (22.39), then followed by Canada (20.73), Belgium (18.02), Netherlands (16.91), and the UK (15.18) (Fig. 2b).

#### H-index

As shown in Fig. 2c, the USA outranked other countries and regions with the most top H-index of 74, followed by Canada (43), the UK (43), China (35), and Germany (33).

### Analysis of global publication

#### Journals

**Fig. 3:**
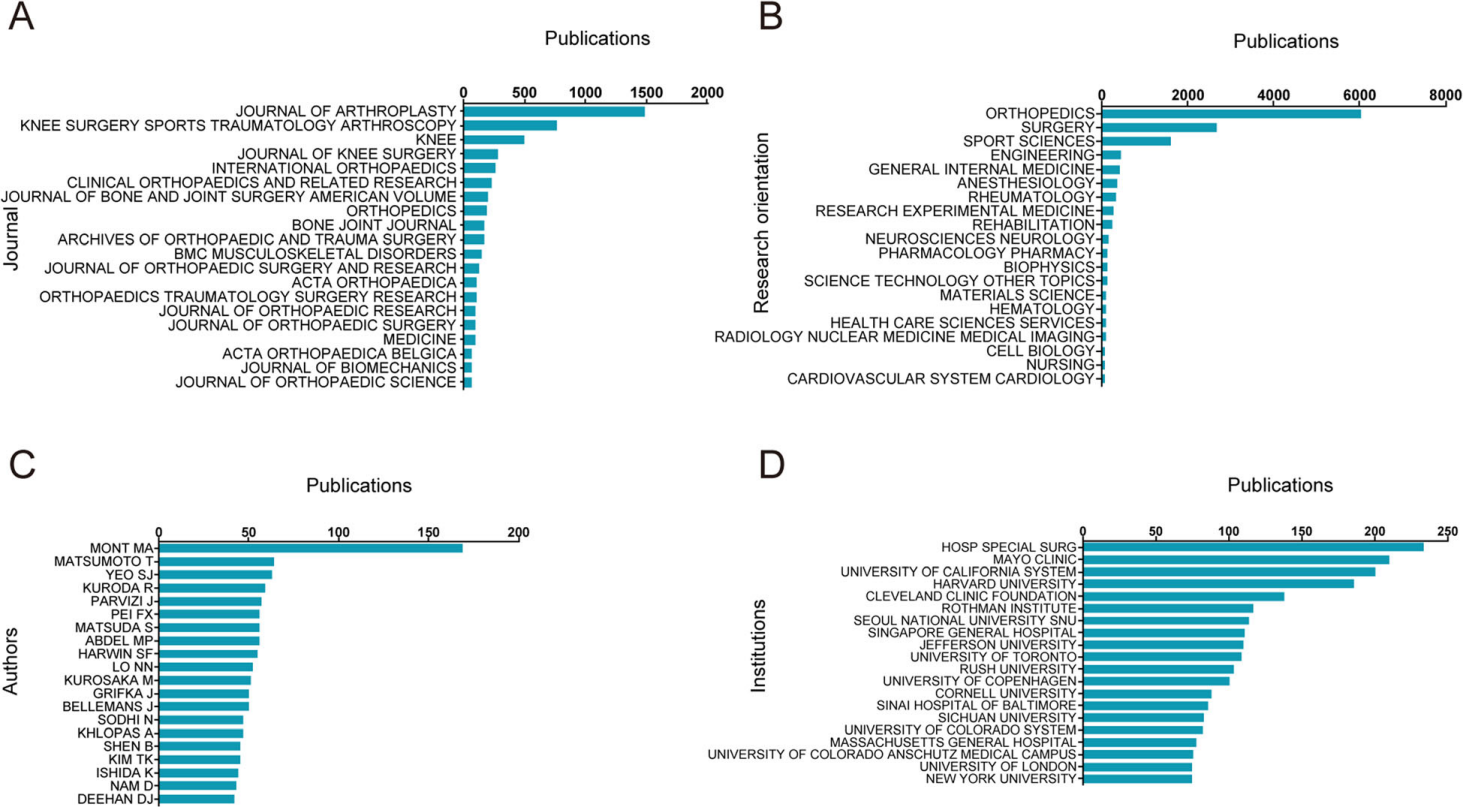
**a** The top 20 journals of publications related to TKA research. **b** The top 20 research orientations and the number of publications in each orientation. **c** The top 20 authors of publications. **d** The top 20 institutions with the highest impact and the number of publications for each institution

*The Journal of Arthroplasty* published 1486 articles/reviews, outranking other journals with the most publications. *Knee Surgery, Sports, Traumatology, Arthroscopy* ranked the second, with 762 publications. Moreover, there were 494 articles/reviews in *The Knee*, 285 in *The Journal of Knee Surgery*, and 258 in *International Orthopaedics* on the TKA field. The top 20 journals with the most publications were presented in Fig. 3a.

#### Research orientation

The top 20 research orientations relevant to TKA were shown in Fig. 3b. The most prevalent areas of research were orthopedics (6013 papers), surgery (2675 papers), sport science (1586 papers), engineering (432 papers), and general internal medicine (418 papers), respectively.

#### Authors

The top 20 authors with the greatest number of related publications were shown in Fig. 3c, who have published a total of 1151 articles/reviews during the last decade, accounting for 13.3% of all literature in this field. Mont MA from the USA outranked other authors with publications of 169 papers, followed by Matsumoto T from Japan with 64 papers, and Yeo SJ from Singapore with 63 papers. It is noteworthy that we included all the authors for analysis in this study, regardless of the authors’ relative contribution towards one single research.

#### Institution output

Hospital special surgery had the greatest number of publications with 234 papers, followed by the Mayo Clinic (210 papers) and then the University of California system (201 papers). Figure 3d presented the top 20 institutions with the most publications.

### Bibliographic coupling analysis

As a well-established method of citation analysis, bibliographic coupling helps create a similarity relationship between documents on the basis of the number of references they share. When two works refer to a common third one in their bibliographies, bibliographic coupling occurs as an indication of sharing a related subject matter for these two works [22]. The VOS viewer was used to analyze the journal names of all publications.

#### Journals

**Fig. 4:**
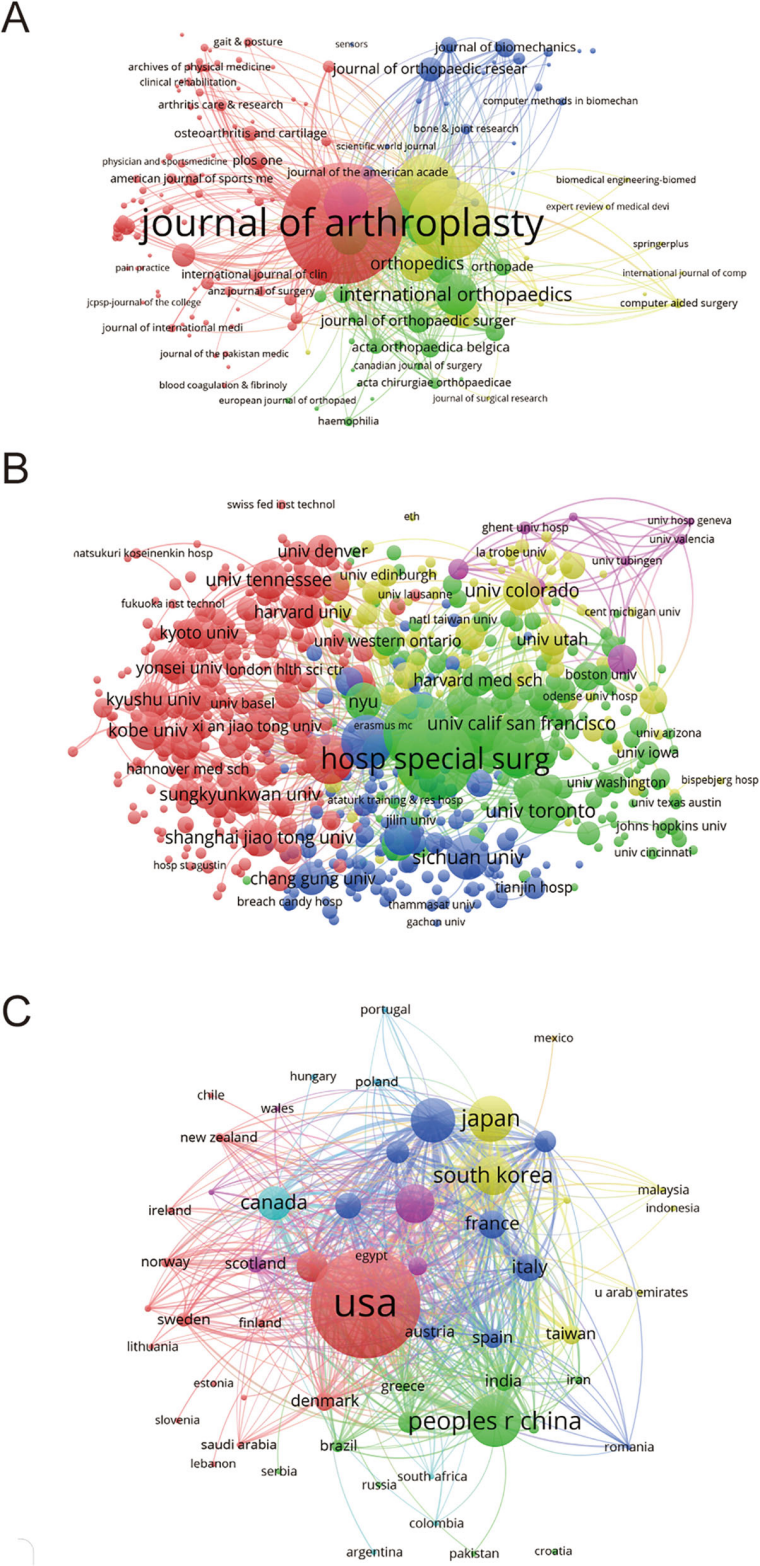
Bibliographic coupling analysis of global research on TKA. **a** Mapping on the 172 included journals in TKA area. **b** Mapping on the 750 institutions on TKA research. **c** Mapping on the 57 countries in this field. The line between two nodes indicates the similarity relationship between the corresponding journals/institutions/countries

As illustrated in Fig. 4a, a total of 172 journals appeared (the minimum number of publications of each journal was over five) in terms of total link strength (TLS). The top five journals with the largest TLS were shown as follows: *The Journal of Arthroplasty* (TLS = 1158621 times); *Knee Surgery, Sports, Traumatology, Arthroscopy* (TLS = 777,852 times); *The Knee* (TLS = 458,865 times); *Clinical Orthopaedics and Related Research* (TLS = 281,285 times); and *International Orthopaedics* (TLS = 271,771 times).

#### Institutions

Papers originating from 750 institutions were analyzed via VOS viewer, and the minimum number of publications from each institution was over five in the bibliometric map (Fig. 4b). The top five institutions with the greatest TLS were as follows: the Mayo Clinic (TLS = 287,724 times), Hospital for Special Surgery (TLS = 242,212 times), Singapore General Hospital (TLS = 181,272 times), Seoul National University (TLS = 145,571 times), and University Colorado (TLS = 139,958 times).

#### Country and regions

There were 57 countries and regions included (the minimum number of publications from each country or region was over five) and all the publications were analyzed by VOS viewer (Fig. 4c). The top five country and regions with largest TLS were presented as follows: the USA (TLS = 1,933,073 times), Germany (TLS = 680,506 times), Japan (TLS = 632,174 times), South Korea (TLS = 631,428 times), and China (TLS = 609,660 times).

### Coauthorship analysis

Coauthorship analysis illustrates the relationship among items based on the number of coauthored documents, which was a powerful tool to assess collaboration trends and to identify leading scientists, countries, and organizations [23].

#### Authors

**Fig. 5:**
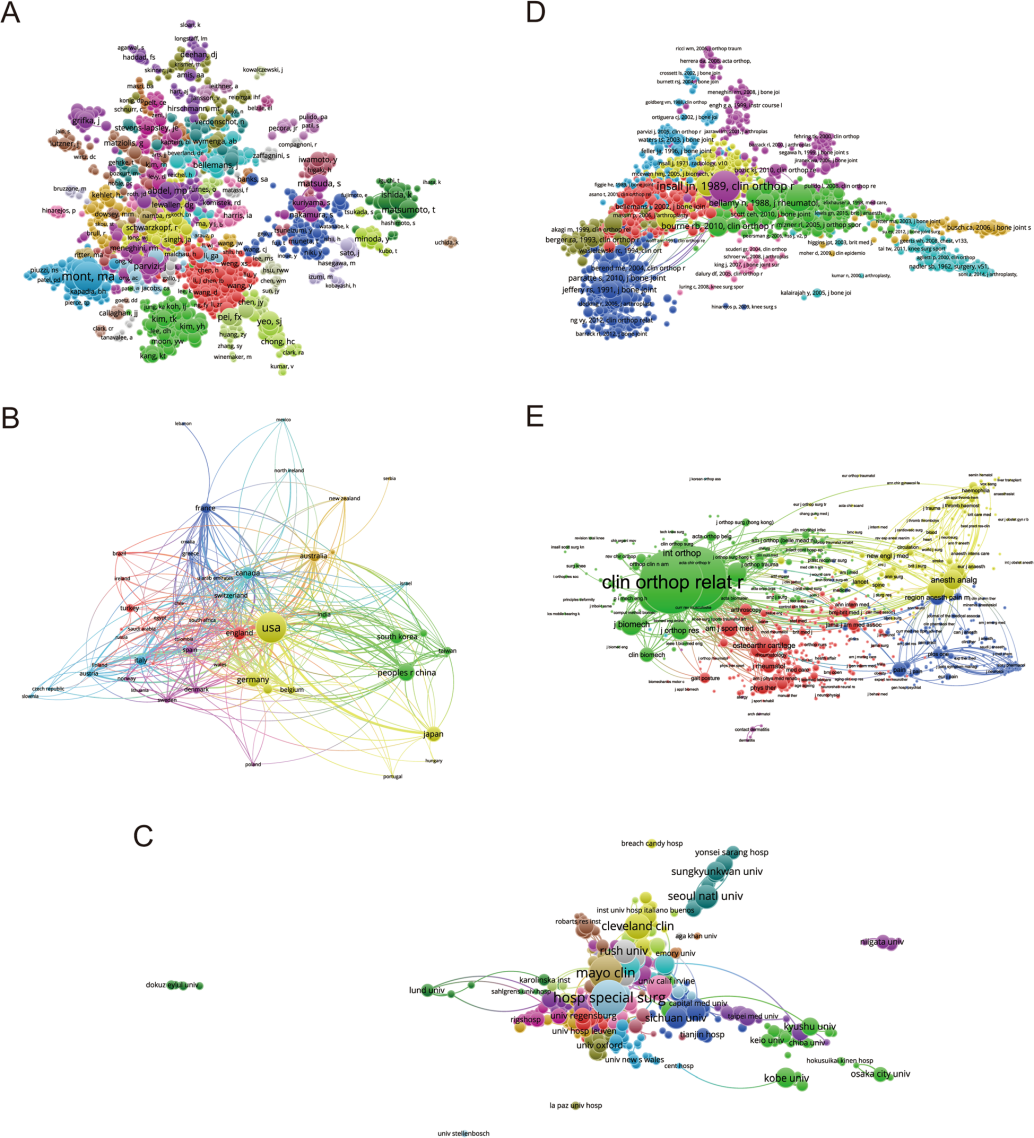
Coauthorship and cocitation analysis of global research on TKA. **a** Mapping of the coauthorship analysis among 1584 identified authors on TKA research. **b** Mapping of 56 identified countries by coauthorship analysis on TKA research. **c** Mapping of coauthorship analysis among 712 institutions in TKA area. **d** Mapping of 2141 included publications by cocitation analysis on TKA research. **e** Mapping on 902 included journals by cocitation analysis in this field

As presented in Fig. 5a, a total of 1584 authors with a minimum limitation of more than 5 publications were identified and analyzed via VOS viewer. The top five authors with greatest TLS were shown as follows: Mont MA (TLS = 768 times), Matsumoto T (TLS = 414 times), Kuroda R (TLS = 400 times), Kurosaka M (TLS = 340 times), and Ishida K (TLS = 325 times).

#### Countries and regions

A total of 56 countries and regions with a minimum limitation of more than 5 publications were identified and analyzed by using VOS viewer (Fig. 5b). The top five country and regions with largest TLS were presented as follows: the USA (TLS = 677 times), the UK (TLS = 318 times), Germany (TLS = 258 times), Canada (TLS = 219 times), and Australia (TLS = 204 times).

#### Institutions

As shown in Fig. 5c, there were 712 institutions finally included with a minimum limitation of more than 5 publications, whose publications were analyzed by VOS viewer. Moreover, the Hospital for Special Surgery (TLS = 224 times), Cleveland Clinic (TLS = 191 times), University Colorado (TLS = 154 times), Mayo Clinic (TLS = 142 times), and University Tennessee (TLS = 128 times) were the top five institutions with greatest TLS.

### Cocitation analysis

Cocitation analysis establishes the relationship of items on the basis of how many times they are cited together and is demonstrated as a way to help identify key literature for cross-disciplinary ideas [24].

#### Publications

A total of 2141 publications were included (the minimum number of citations for one reference was over 20 times) and analyzed via VOS viewer (Fig. 5d). The top five publications with largest TLS were presented as follows: Insall et al. [25] (TSL = 13,413 times), Kurtz et al. [26] (TSL = 8451 times), Ewald et al. [27] (TSL = 7568 times), Bourne et al. [28] (TSL = 6666 times), and Berger et al. [29] (TSL = 6571 times).

#### Journals

The journal was included if the minimum number of citations from one source was over 20 times. In total, there were 902 journals which met the aforementioned criteria (Fig. 5e). The top five journals with greatest TLS were illustrated as follows: *Clinical Orthopaedics and Related Research* (TLS = 941,132 times), *The Journal of Arthroplasty* (TLS = 900,527 times), *The Journal of Bone and Joint Surgery American Volume* (TLS = 643,757 times), *The Journal of Bone and Joint Surgery British Volume* (TLS = 398,451 times), and *Knee Surgery, Sports, Traumatology, Arthroscopy* (TLS = 310,785 times).

#### Co-occurrence analysis

**Fig. 6:**
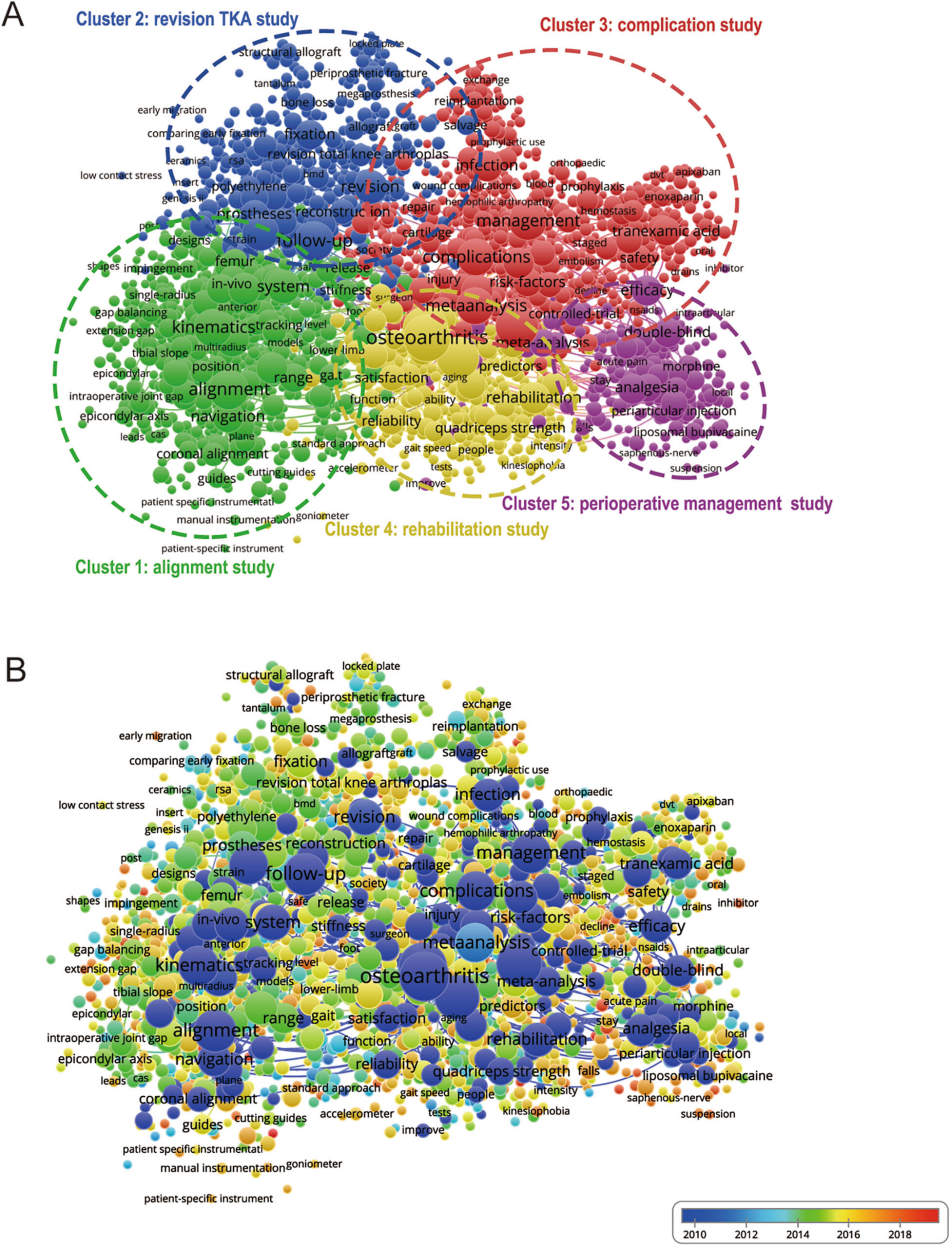
Co-occurrence analysis on TKA research. **a** Mapping of keywords on TKA research; the size of nodes represents the frequency, while the lines between nodes reflect the co-occurrence relationship. A total of 2225 included keywords are divided into 5 clusters: alignment study (color green), revision TKA study (color blue), complication study (color red), rehabilitation study (color yellow), and perioperative management study (color purple). **b** Distribution of keywords according to the frequency of appearance. The color blue indicates the keywords appear earlier, whereas the color red reflects the later occurrence

Co-occurrence analysis illustrates the relationship of items based on the number of publications where they occur together [30]. Not only the popular areas and research directions could be identified through co-occurrence analysis, but also it turns out to be an important indicator to monitor developments in scientific areas and other disciplines. The keywords, which were used over five times among included publications, were identified and analyzed via VOS viewer. As presented in Fig. 6a, the 2225 included keywords could be divided into approximately 5 clusters: “alignment study”, “revision TKA study”, “complication study”, “rehabilitation study”, and “perioperative management study” (Fig. 6a). The most prominent topics about TKA research were shown as follows. In the cluster of “alignment study”, the main keywords were alignment, kinematics, flexion, and system. As for the “revision TKA study” cluster, the primary keywords were follow-up, prosthesis, revision, and fixation. In the “complication study” cluster, the most used keywords were surgery, meta-analysis, management, and complications. As for the “rehabilitation study” cluster, the prominent keywords were osteoarthritis, pain, rehabilitation, and quadriceps strength. In the cluster of “perioperative management study”, the frequently used keywords were efficacy, post-operative pain, double-blind, and femoral nerve block.

In Fig. 6b, different colors were applied by VOS viewer for each keyword, based on the mean times they appeared in the all included publications. The color blue indicated the keywords appeared earlier, whereas the color red stood for the later occurrence. As illustrated in Fig. 6b, a trend of balanced development existed in all the five clusters respectively during the past decade. By comparison, more studies focused on the cluster of “revision TKA” after 2014. However, recent trends indicated that the other four clusters were also undergoing different degrees of changes on the research hotspot, which meant a diversified developing trend.

## Discussion

### Global trends in TKA research

Bibliometric and visualized analysis is considered as valid tools for describing the current status and predicting the future development trends about the research of interest. Therefore, we aim to evaluate TKA research through bibliometric and visualized analysis and further illustrate its future global trends from the aspects of publications, contributing countries, institutions, and the research orientations. In the past decades, great development happened in the evolving field of TKA research. As shown in this study, the number of publications has steadily increased year by year. In addition, the RRIs of publications had also risen dramatically in the meantime. A total of 86 country and regions have published papers in this domain, which indicated a potential increase of studies which focus on TKA research and offer in-depth knowledge in the next few years.

### Quality and status of global publications

The total number of citations and the H-index reflect the quality of the publications and academic impact of one country [31]. According to our study, the USA outranked the other countries in terms of the total number of publications and citations, as well as H-index, making the largest contribution to global TKA research. The UK and Canada also contributed a lot with a considerable total citation frequency and H-index, especially for Canada, which ranked second in the average citation frequency. Nevertheless, some countries such as Denmark, Canada, and Belgium have also played an important role when considering their high average citation frequencies. It was worth noting that developing countries also made crucial contributions. For instance, China ranked among top five with regard to the total number of publications and citations, along with H-index. Due to the high incidence of knee arthritis, large population base and subsequent great numbers of TKAs, developing countries therefore made enormous investments in the field of TKA research, which might partially explain this result.

As for journals, *The Journal of Arthroplasty*, *Knee Surgery, Sports, Traumatology, Arthroscopy* and *The Journal of Knee Surgery* made great contributions which published the most studies on TKA. According to this trend, the listed journals in Fig. 4a may continue the “main channel” for future findings in this field.

Almost all of the top 20 institutions were from the top five countries with the largest publication numbers, and more than half of them were located in the USA, once again reflecting the great academic impact of the USA in this area. This indicated the significant role of these first-class institutions in improving the academic level of a country. Mont MA, Matsumoto T, and Yeo SJ were the top three authors with the most publications in TKA. The top 20 authors listed in Fig. 3c represented the research pioneers who may substantially influence the direction of future studies. Therefore, in order to grasp the latest advancements in this filed, more attention should be paid on their work with a relatively high priority.

In this study, bibliographic coupling analysis was used to illustrate the similarity relationship between the identified publications from the aspects of journal, institution, and country, respectively. Bibliographic coupling occurs when two studies share with the common references in their bibliographies, so that it can provide a deep, direct insight on the links among these relevant literature and further interpret how these links are built and used by the authors. According to our data, *The Journal of Arthroplasty* was the most closely related journal, and the Hospital for Special Surgery published the most relevant papers. As for the countries, the USA outshined the rest of the countries which maintained its leading position in the domain of TKA research. Coauthorship analysis was utilized to evaluate the collaboration between different authors, countries, and institutions. Research object (author/country/institution) with higher TLS was considered more likely to be cooperative with others. In this study, the results of coauthorship analysis were Mont MA, the USA, and the Hospital for Special Surgery, respectively. Cocitation analysis was conducted to illustrate the influence of studies, by counting how many times they were cited together. In our study, *Clinical Orthopaedics and Related Research* was the journal with the highest citation frequency, which could be considered as the landmark studies about the TKA research.

### Research focus on TKA

Co-occurrence analysis was based on the number of publications with the common occurrence, so as to evaluate the relationship among identified items. Furthermore, it was considered as an efficient way to predict the future trends and hotspots in the research field of interest. In our study, we presented a network map of co-occurrence relationship by analyzing the keywords of all the included studies. Eventually, a total of five possible research orientations were summarized as follows: “alignment study,” “revision TKA study,” “complication study,” “rehabilitation study,” and “perioperative management study” (Fig. 6a). With the help of this network map, we had chances to further clarify the developing trends in the future. As shown in the co-occurrence map, such keywords as osteoarthritis, alignment, kinematics, follow-up, surgery, and management, were highlighted with bigger icons, which almost evenly distributed in the orientations of “alignment study,” “revision TKA study,” “complication study,” and “rehabilitation study.” Thus, the investment and requirement of high-quality studies are stilled necessary within the context of these five research directions, especially for the first four aforementioned orientations.

The overlay visualization map, with items which were noted with different color according to the average time when the keywords occurred [32], was kind of similar to the co-occurrence map. However, it could more directly monitor the progress of the research and predict the popular topics in the future. In this overlay visualization map (Fig. 6b), different colors represented the relevant year of publication. From the results, “revision TKA” accounted for larger proportions for color yellow and red, which indicated more studies focused on the research of “revision TKA” after 2014. This is probably because of the inherent occurring order between primary TKA and revision TKA. Nevertheless, nodes of various colors (from blue to red) could all be found with considerable densities in the five clusters, which meant a trend of balanced development existed in these five research directions respectively during the past decade. Furthermore, each direction itself was also undergoing the changes of research hotspot, which indicated a diversified developing trend.

In light of our findings in this study, the number of publications related to TKA research has been rapidly growing since 2010. Therefore, TKA continues to thrive and play an important part in managing severe knee arthritis, which is served as the effective surgical therapy for end-term arthritis. This blossoming result will in turn encourage more and more researchers to devote to future study. Through the bibliometric and visualized analysis, investigators could have a vivid overall impression of this field, including the leading countries, authors, and institutions, along with the partnership and academic impact of them. With this information, a transparent “main channel” has been created for the investigators, so that they could selectively obtain advanced knowledge and valuable discoveries according to their own requirement. Furthermore, the co-occurrence analysis could depict the developing trends and research hotspots in this area, which may further provide the researchers with inspirations of topic selection and help the funding agencies make profit investment plans.

### Strength and limitations

To the best of our knowledge, this is the first study which conducted bibliometric analysis on TKA research. To better comprehensively capture the current status and future trends of researches on TKA, bibliometric and visualized analysis were used to identify the hotspots and collaborative relationship among different countries, authors, and institutions. However, this study inevitably has some limitations. Firstly, although the included publications were adequate enough to reflect the current status, we only retrieved data from WoS database. Thus, we may omit some publications due to database bias. Secondly, almost all of the identified publications were in English, some relevant articles may not have been included due to language bias [33, 34]. Thirdly, there are intrinsic differences between the results of bibliometric analysis and real-world study. For instance, some relatively new publications with high quality might not attach enough attention due to lower citation frequency. Last, but not the least, bias may still exist when considering the same short name or various expressions of some authors and keywords.

## Conclusions

In this study, the current status and global trends of TKA research were delineated through bibliometric analysis. The USA has made tremendous contribution to this area, establishing its leading position of the global research on TKA. It can be predicted that an increasing number of papers would be published in the next few years, which indicates an underlying vigorous development of TKA research. In particular, a trend of balanced development may exist in the field of TKA, accompanied with inherent changes of hotspots in each sub-orientation.

## Data Availability

All data generated or analyzed during this study are included in this published article (and its supplementary information files).

## Abbreviations

TKA: Total knee arthroplasty
OA: Osteoarthritis
WoS: Web of Science
SCIE: Science Citation Index-Expanded
RRI: Relative research interest
